# Perceived Weight Discrimination Mediates the Prospective Relation Between Obesity and Depressive Symptoms in U.S. and U.K. Adults

**DOI:** 10.1037/hea0000426

**Published:** 2016-10-17

**Authors:** Eric Robinson, Angelina Sutin, Michael Daly

**Affiliations:** 1 Department of Psychological Sciences, Institute of Psychology, Health & Society, University of Liverpool; 2 Florida State University College of Medicine; 3 Behavioural Science Centre, Stirling Management School, University of Stirling and UCD Geary Institute, University College Dublin

**Keywords:** obesity, depression, obesity stigma, discrimination, weight stigma

## Abstract

***Objective:*** Obesity has been shown to increase risk of depression. Persons with obesity experience discrimination because of their body weight. Across 3 studies, we tested for the first time whether experiencing (perceived) weight-based discrimination explains why obesity is prospectively associated with increases in depressive symptoms. ***Method:*** Data from 3 studies, including the English Longitudinal Study of Ageing (2008/2009–2012/2013), the Health and Retirement Study (2006/2008–2010/2012), and Midlife in the United States (1995/1996–2004/2005), were used to examine associations between obesity, perceived weight discrimination, and depressive symptoms among 20,286 U.S. and U.K. adults. ***Results:*** Across all 3 studies, Class II and III obesity were reliably associated with increases in depressive symptoms from baseline to follow-up. Perceived weight-based discrimination predicted increases in depressive symptoms over time and mediated the prospective association between obesity and depressive symptoms in all 3 studies. Persons with Class II and III obesity were more likely to report experiencing weight-based discrimination, and this explained approximately 31% of the obesity-related increase in depressive symptoms on average across the 3 studies. ***Conclusion:*** In U.S. and U.K. samples, the prospective association between obesity (defined using body mass index) and increases in depressive symptoms in adulthood may in part be explained by perceived weight discrimination.

There is convincing evidence for a bidirectional link between obesity and depression (de Wit et al., 2010; Luppino et al., 2010): Depression is associated with future weight gain (Grundy, Cotterchio, Kirsh, & Kreiger, 2014; Luppino et al., 2010), and persons with obesity are at greater risk of developing depressive symptoms than are their “normal” weight counterparts (Faith et al., 2011; Herva et al., 2006; Roberts, Deleger, Strawbridge, & Kaplan, 2003). There is evidence that the severity of obesity predicts the strength of the association between obesity and depression, whereby persons with Class II obesity and above are most likely to suffer from depressive symptoms (Onyike, Crum, Lee, Lyketsos, & Eaton, 2003; Preiss, Brennan, & Clarke, 2013; Vogelzangs et al., 2010). Although the prospective relation between obesity and depression has now been confirmed, the mechanisms explaining why persons with obesity are at an increased risk of developing depressive symptoms remain unclear (Luppino et al., 2010; Preiss et al., 2013). Moreover, the majority of studies that have examined potential mechanisms linking obesity to depression have relied on cross-sectional designs and/or nonrepresentative samples (Preiss et al., 2013).

A number of studies have shown that obesity is stigmatized, and a substantial portion of persons with obesity report being treated unfairly because of their weight, otherwise known as *perceived weight discrimination* (Jackson, Steptoe, Beeken, Croker, & Wardle, 2015b; Puhl & Heuer, 2009; Sutin & Terracciano, 2013). Recent findings have linked experiencing weight-based discrimination with a variety of adverse health outcomes. For example, individuals who report experiencing discrimination because of their weight are more likely to suffer ill health as indexed by both self-report and physiological measures (Chen et al., 2007; Fettich & Chen, 2012; Sutin, Stephan, Carretta, & Terracciano, 2015; Sutin, Stephan, Luchetti, & Terracciano, 2014). Moreover, perceived weight discrimination is most common among persons with Class II obesity and above, among whom risk of future depression is highest (Dutton et al., 2014; Jackson et al., 2015b; Spahlholz, Baer, Konig, Riedel-Heller, & Luck-Sikorski, 2016). For example, recent data from a representative survey of German participants indicate that 19% and 38% of participants with Class II and Class III obesity report experiencing weight-based discrimination (Sikorski, Spahlholz, Hartlev, & Riedel-Heller, 2016). In addition, a number of theoretical models suggest that experiencing weight discrimination is likely to act as a form of psychological stressor (Major, Eliezer, & Rieck, 2012; Tomiyama, 2014), which could reduce self-worth and increase negative affect among persons with obesity (Crocker, Cornwell, & Major, 1993; Sikorski, Luppa, Luck, & Riedel-Heller, 2015). Thus, the experience of weight-based stigma may be an important factor explaining why obesity is associated with increased depressive symptoms.

A recent cross-sectional study of English older adults showed that perceived weight discrimination is associated with lower quality of life and more depressive symptoms (Jackson, Beeken, & Wardle, 2015a). Although cross-sectional studies that link weight-based discrimination to adverse psychological outcomes are informative, they are also limited as it is plausible that reverse causality may explain these associations; those suffering from depression may be particularly likely to perceive weight-based discrimination (Jackson et al., 2015a), which has been shown to further propagate weight gain (Sutin & Terracciano, 2013). To date, there have been no examinations of the prospective associations among obesity, perceived weight discrimination, and depression. The aim of the current research was to examine whether experiencing (perceived) weight-based discrimination mediates the prospective association between obesity and subsequent changes in depressive symptoms in three large cohort studies of U.S. and U.K. adults. We predicted that experiencing weight discrimination would in part explain why persons with obesity show increases in depressive symptoms over time. A further aim of the current research was to examine whether gender moderated this effect. We reasoned that women may be more likely to experience increases in depressive symptoms as a result of experiencing weight-based discrimination because of the importance attached to female thinness in our current social climate (Thompson & Stice, 2001).

## Study 1: English Longitudinal Study of Ageing (ELSA)

Our first aim was to make use of data from the ELSA to examine whether there is evidence that perceived weight discrimination mediates the prospective association between obesity and depressive symptoms among older U.K. adults.

### Sample

Participants were drawn from ELSA, an ongoing prospective cohort study established in 2002 to study the health and ageing of community dwelling older adults (≥50 years). The initial ELSA sample was recruited from three waves of the Health Survey for England (1998, 1999, 2001), an annual cross-sectional survey based on a stratified random sample of English households. Interview data are collected every 2 years, and a clinical assessment is conducted every 4 years. In the current analyses, we calculate body mass index (BMI) from height and weight measurements collected as part of the Wave 4 (2008–2009) health assessment and examine longitudinal change in depressive symptoms over the 4-year period from Wave 4 to Wave 6 (2012–2013). Participants completed a measure of discrimination as part of the Wave 5 (2010–2011) interview. To be included in the current analyses, participants needed to have provided complete demographic, body mass index (BMI), and depressive symptom data as well as the perceived weight discrimination measure (*N* = 6,000). Sample characteristics are detailed in Table 1. Participants in all three studies provided informed consent and ethical approval was obtained for each study.[Table-anchor tbl1]

### Measures

#### BMI

As part of the Wave 4 health assessment, trained nurses weighed participants to the nearest 0.1 kg using Tanita THD-305 portable electronic scales. Standing height was measured to the nearest millimeter using a portable stadiometer. Participants stood on the center of a baseplate looking straight ahead in order to gauge height accurately and consistently. BMI was derived as kg/m^2^ and defined as normal weight (BMI <25), overweight (BMI 25–29.9), Class I (BMI 30–34.9), Class II (BMI 35–39.9), and Class III obesity (BMI 40 and above).

#### Perceived weight discrimination

In all three studies, participants completed an adapted version of the Perceived Everyday Experiences With Discrimination Scale (Williams, Yan, Jackson, & Anderson, 1997). Participants first reported how frequently they perceived a set of discriminatory experiences to occur in their day-to-day lives. During Wave 5 of ELSA, the frequency of five forms of unfair treatment was assessed (“you are treated with less respect or courtesy,” “you are threatened or harassed,” “you receive poorer service than other people in restaurants and stores,” “people act as if they think you are not clever,” “you receive poorer service or treatment than other people from doctors or hospitals”) on a 6-point scale ranging from *never* to *almost every day*. Next, participants who reported having experienced discrimination in daily life were asked to select the reason(s) they believed they were discriminated against from a list that included weight. Participants could choose as many or as few attributions for the unfair treatment as necessary. In fitting with other studies that have examined the association between perceived weight discrimination and health outcomes (Jackson et al., 2015a; Sutin et al., 2015), perceived weight discrimination (dichotomous variable) was defined as those who reported experiencing discrimination and indicated they believed that weight was a reason for this discrimination. Rates of perceived weight discrimination across body weight categories are detailed in Table 2.[Table-anchor tbl2]

#### Depressive symptoms

A validated eight-item version of the Center for Epidemiology Depression Scale (CES-D; Radloff, 1977; Turvey, Wallace, & Herzog, 1999) was administered to assess depressive symptoms at baseline and at follow-up. The short form CES-D uses a *yes/no* response format to assess feelings over the last week, including sadness, lethargy, loneliness, happiness, and enjoyment of life. Positively worded items were reverse scored, and a total sum score was generated ranging from 0 to 8, with higher scores indicating greater depressive symptoms. The CES-D demonstrated sufficiently high levels of reliability (Cronbach’s α = .79 in both waves) and a moderate degree of stability across study waves (*r* = .50, *p* < .001).

#### Covariates

We based our choice of covariates on recorded variables likely to be associated with depression and/or obesity (Preiss et al., 2013; Luppino et al., 2010). Participants reported demographic information at baseline (Wave 4, 2008–2009) including their age, gender, ethnicity (White vs. other), education level (1 = *no qualifications*, 7 = *degree level qualification or above*), marital status (married, cohabiting, other), and employment status (employed/self-employed, unemployed, homemaker, retired, permanently sick or disabled). Participants also reported details relating to their health and health behavior. Specifically, participants indicated whether they had a longstanding illness, whether they were a current smoker, the frequency of their alcohol consumption in the last week (0 = *drank on none of the last 7 days*, 7 = *drank on all days in the last week*), and the frequency they engage in moderate and vigorous physical activity (1 = *more than once a week*, 4 = *hardly ever, or never*).

#### Mediation analyses

Across all three studies, mediation analysis was used to identify whether weight status at baseline (i.e., overweight, Class I, II, and III obesity relative to normal weight) had an indirect effect on depressive symptoms (standardized to have a mean of 0 and a standard deviation of 1) at follow-up through perceived weight discrimination. All mediation analyses were adjusted for initial depressive symptoms and covariates that may confound the relationship between obesity and depression: age, age^2^ (to account for a potential nonlinear relationship), gender, education, marital status, and employment status. We first established the preconditions necessary for successful mediation (Baron & Kenny, 1986). This involved establishing an association between (a) weight status categories and depressive symptoms (total effect, path *c*), (b) weight status categories and perceived weight discrimination (path *a*), and (c) perceived weight discrimination and depressive symptoms (path *b*) in a model that included baseline weight status. When the conditions for mediation were met, we conducted further analyses of the potential indirect effects (path *a* × *b*) identified using the *khb* command in STATA (Version 13; Karlson, Holm, & Breen, 2012; Kohler, Karlson, & Holm, 2011). We used this method because our perceived weight discrimination mediator variable was dichotomous, and path *a* coefficients (independent variable to dichotomous mediator) derived from logistic regression cannot be multiplied directly with the ordinary least squares path *b* coefficients (dichotomous mediator to continuous dependent variable, path *b*) using the standard product of coefficients approach (Preacher & Hayes, 2008). The *khb* method decomposes the total effect of obesity on depression into a direct effect and an indirect effect through perceived weight discrimination. It also provides estimates of the magnitude and statistical significance level of the indirect effect and proportion of the total association accounted for by this pathway.

#### Robustness tests

We conducted supplementary mediation analyses in which each model was adjusted for health behavior and health status. We considered this an additional stringent test of the study hypotheses, given that health-related variables may act as either confounding factors and/or additional pathways from perceived discrimination to depressive symptoms. If including these variables in our regressions did not notably change the indirect association between obesity and depressive symptoms through perceived discrimination, we considered the relationship to be unlikely to be affected by health-related variables. We also tested whether the mediation results were notably different if a continuous measure of body weight (i.e., BMI) was used as the predictor variable or if a dichotomous indicator of clinically significant depression was used as the outcome measure. Specifically, we tested whether weight discrimination mediated the longitudinal association between BMI (treated continuously) and changes in depressive symptoms and whether weight discrimination explained the link between weight categories and changes in the presence of clinically significant depression levels over time. For the latter analyses, we used scale specific cut-off scores for clinically significant depression scores to identify those meeting the criteria for depression (see Table S1 in the online supplemental materials for scale cut off scores in each study and depression rates).

### Results and Conclusion

Participants in the Class II and III obesity categories were at an increased risk of developing more depressive symptoms from baseline to follow-up (*p* < .01), as shown in Table 3. As expected, the proportion of participants experiencing weight discrimination increased markedly across weight categories (i.e., overweight, obesity Classes I, II, III; see Table 2). For example, among normal weight and overweight participants, fewer than 1% reported weight discrimination, whereas >20% of Class II and III obese participants reported experiencing weight discrimination. Perceived weight discrimination was found to be a significant predictor of increased depressive symptoms from baseline to follow-up (β = .188, *p* < .001) in models adjusting for weight status at baseline, as outlined in Table 3.[Table-anchor tbl3]

We found a significant indirect effect between Class II (β = .036, *SE* = .012, *p* < .01, 95% CI = .013–.059) and Class III obesity (β = .057, *SE* = .019, *p* < .01, 95% CI = .020–.095) and longitudinal change in depressive symptoms through perceived weight discrimination, as shown in Table 3. In total, 18.1% of the total effect of Class II obesity and 20.6% of the effect of Class III obesity on depressive symptoms was mediated through perceived weight discrimination. Our robustness tests indicated that perceived weight discrimination explained approximately 28% of the association between Class II and III obesity and depressive symptoms in models adjusting for the presence of a longstanding limiting illness, whether the participant smoked, and the frequency with which the participant drank and exercised (see Table S2 in the online supplemental materials). We interpret this as evidence that the contribution of perceived weight discrimination to explaining the obesity–depression link is unlikely to be due to confounding by health or health behavior in this study.

In addition, we found that 22.9% of the total effect of BMI (continuous variable) on increases in depressive symptoms (B = .011, *SE* = .002, *p* < .01) was mediated by weight discrimination (B = .002, *SE* = .0001, *p* < .01), as shown in Table S3 of the online supplemental materials. Weight discrimination predicted increases in clinically significant depression levels over time (OR = 1.51, *p* < .05, 95% CI = 1.04–2.19) and mediated 22.3% of the link between Class II and Class III obesity and clinically significant depression on average, as shown in Tables S4 and S5 of the online supplementary materials. These supplementary analyses show that the role of perceived weight discrimination in mediating the link between body weight and depression is not markedly different from our main analyses when either a continuous BMI measure or a dichotomous measure of clinically significant depression was used.

## Study 2: Health and Retirement Study (HRS)

In Study 1, we found evidence that the relation between obesity and depressive symptoms is mediated by perceived weight discrimination among older English adults. A potential limitation of Study 1 was that the mediator variable (perceived weight discrimination) was measured after the baseline measures of BMI and depression. We were able to address this in Study 2. Moreover, given that the relation between obesity and depression has been suggested to be particularly strong among Americans (Luppino et al., 2010), in Study 2 we aimed to replicate the findings of Study 1 in a large sample of older U.S. adults.

### Sample

A total of 9,908 participants were drawn from the HRS, a longitudinal study of Americans over the age of 50 and their spouses. In 2006, HRS implemented an enhanced face-to-face interview that included a standardized measurement of weight and height and a psychosocial questionnaire that participants completed at home and mailed back to the University of Michigan. Half of the HRS sample participated in the enhanced interview in 2006; the other half participated in 2008. These two samples were combined as baseline. Participants completed the same assessment again 4 years later, in 2010 and 2012, respectively. These assessments were combined as the follow-up to give each participant a 4-year follow-up interval. See Table 1 for sample demographic information.

### Measures

#### BMI

As part of the enhanced face-to-face interview, trained staff measured and weighed participants. BMI was derived as kg/m^2^ and categorized into categories as in Study 1.

#### Perceived weight discrimination

Participants completed the Perceived Everyday Experiences With Discrimination Scale as described in Study 1 (Williams et al., 1997) at baseline.

#### Depressive symptoms

At baseline and follow-up, participants completed a short version of the CES-D scale (Turvey et al., 1999). Participants rated nine items (*yes/no*) that measured depressive symptoms during the last week (e.g., “I felt depressed”), which were summed for a total depressive symptoms score.

#### Covariates

Demographic information was provided at baseline (2006–2008) and included age, age^2^, gender, ethnicity (White vs. other), years of education, marital status (married, separated/divorced, widowed, never married), and employment categories (employed, unemployed, homemaker, retired, temporary leave, disabled). Health and health behavior were assessed using a measure of disease burden at baseline (a sum of eight diagnosed chronic conditions), history of ever smoking, frequency of vigorous physical activity, and average alcohol consumption in a week over the last 3 months.

### Results and Conclusion

We used the same analysis strategy as in Study 1. In an initial model unadjusted for perceived weight discrimination, individuals of Class I, II and III obesity were at an elevated risk of increased depressive symptoms from baseline to follow-up, as detailed in Table 4. The numbers of participants experiencing weight discrimination increased as BMI increased. For example, among normal weight and overweight participants around 2% reported experiencing weight discrimination, whereas >20% of Class II and III obese participants reported weight discrimination (see Table 2). Those who reported perceived weight discrimination showed a significant increase in depressive symptoms over the 4-year period from baseline to follow-up (β = .141, *p* < .001), as shown in Table 4. We observed significant indirect effects of obesity Classes I (β = .011, *SE* = .003, 95% CI = .005–.016, *p* < .01), II (β = .026, *SE* = .006, 95% CI = .013–.038, *p* < .01) and III (β = .046, *SE* = .011, 95% CI = .024–.069, *p* < .01) on depressive symptoms through perceived weight discrimination. Effect ratios showed that perceived weight discrimination explained approximately 34% of the effect of Classes I, II, and III obesity on longitudinal changes in depressive symptoms, as shown in Table 4.[Table-anchor tbl4]

#### Robustness tests

As in Study 1, we also tested the effect of perceived weight discrimination on the relation between obesity and change in depressive symptoms while controlling for other health and health behavior variables (i.e., disease burden, physical activity, smoking and alcohol consumption). This analysis confirmed that perceived weight discrimination significantly mediated the relation between obesity (Classes I, II, and III) and change in depressive symptoms while controlling for a range of potential confounding variables, explaining approximately 35% of this association (see Table S2 of the online supplemental materials). As in Study 1, we found that weight discrimination explained a substantial portion (38.6%) of the longitudinal link between BMI (continuous variable) and increases in depressive symptoms (total effect: B = .005, *SE* = .001, *p* < .01; indirect effect: B = .002, *SE* = .0004, *p* < .01), as shown in Table S6 of the online supplemental materials. Once again, weight discrimination predicted increases in the presence of clinically significant depression from baseline to follow-up (*OR* = 1.50, *p* < .01, 95% CI = 1.22–1.84) and partially mediated of the link between Class I, II, and III obesity and clinically significant depression (26.4% explained on average), as shown in Tables S4 and S7 of the online supplemental materials.

## Study 3: Midlife in the United States (MIDUS)

In the third study we sought to replicate the findings of Study 1 and Study 2 in a sample with a more diverse age range.

### Sample

Data were drawn from the MIDUS study, a national longitudinal study of the psychosocial factors that influence the health and well-being of Americans from midlife to old age (for comprehensive sample information see Brim, Ryff, & Kessler, 2004). The main sample was recruited via random digit dialing and the total sample includes siblings within recruited households and a sample of twin pairs. In total 7,108 noninstitutionalized adults aged 25 to 74 were first interviewed in 1995 and 1996. Those included in the current analyses needed to have provided complete demographic information and to have completed both the baseline discrimination measure and a measure of depression at baseline (1995) and follow-up 10 years later (2004 through 2005). Demographic data for those individuals (*N* = 4,283) who met these criteria and were included in the sample are outlined in Table 1.

### Measures

#### BMI

Participants reported their height and weight as part of the MIDUS baseline survey. As in Studies 1 and 2, BMI was derived as kg/m^2^ and divided into overweight, obesity Classes I, II, and III categories. Self-reported BMI and objectively verified BMI recorded during a physical exam were available for a subset of 900 MIDUS participants and found to be highly correlated in this sample (*r* = .92, *p* < .001; Robinson, Hunger, & Daly, 2015).

#### Perceived weight discrimination

Weight discrimination was derived from the measure of everyday discrimination as in Studies 1 and 2 (Williams et al., 1997). At baseline and follow-up, participants were asked to indicate how frequently they experienced nine forms of discriminatory treatment, which included similar items to those used in Study 1 and Study 2 (“you are treated with . . . less courtesy than other people,” “. . . less respect than other people,” “you receive poorer service than other people,” “people act as if they . . . think you are not smart,” “. . . are afraid of you,” “. . . think you are dishonest,” “. . . think you are not as good as they are,” “you are . . . called names or insulted,” “. . . threatened or harassed”). After making these ratings, participants were asked to select the reason(s) for this discrimination from a list, including “weight or height.” Perceived weight discrimination (dichotomous variable) was defined as those who identified weight or height as a reason for having experienced discrimination.

#### Depressive symptoms

The World Health Organization Composite International Diagnostic Interview-Short Form (Kessler, Andrews, Mroczek, Ustun, & Wittchen, 1998) was used to gauge the presence of depressive symptoms at baseline and follow-up. Participants first indicated if they “felt sad, blue, or depressed” or “lost interest in most things” for 2 weeks in the last 12 months. Those who endorsed either of these items then responded to seven (*yes/no*) follow-up questions assessing depressive symptoms relating to how they felt during this period (e.g., “feel down in yourself, no good, or worthless”). A rating was derived from the two measures, ranging from 0 to 7 (0 = *no 2-week period of depressed affect or anhedonia in the last year*, 7 = *highest depressive symptom score*).

#### Covariates

Additional covariates included age, age^2^, gender, ethnicity (White vs. other), educational level (1 = *no school/some grade school*, 12 = *PhD/MD level*), marital status and (married, separated, divorced, widowed, never married), and employment status (employed, self-employed, unemployed, laid off, homemaker, student, retired, on leave, permanently disabled, other). Health and health behavior were gauged by the presence of a chronic health condition at baseline, current regular smoking, the frequency of moderate and vigorous physical activity in the last month, and alcohol consumption in the last month.

### Results and Conclusion

We used the same analysis strategy as in Studies 1 and 2. In the first model unadjusted for perceived weight discrimination, depressive symptoms among individuals of Class II and III obesity increased from baseline to follow-up 10 years later (see Table 5). Once again, perceived weight discrimination increased markedly in line with weight status, as shown in Tables 2 and 5. Perceived weight discrimination was a significant predictor of increased depressive symptoms from baseline to follow-up (β = .152, *p* < .001), and the inclusion of perceived weight discrimination reduced the strength of the associations between Classes II and III obesity and depressive symptoms at follow-up (see Table 5). Mediation analyses confirmed significant indirect effects of Class II (β = .052, *SE* = .017, 95% CI = .018–.086, *p* < .01) and Class III (β = .081, *SE* = .026, 95% CI = .028–.132, *p* < .01) obesity on depressive symptoms through perceived weight discrimination. An examination of the effect ratios indicated that perceived weight discrimination explained over 31% of the total effect of obesity (Classes II and III) on depressive symptoms.[Table-anchor tbl5]

#### Robustness tests

As in Studies 1 and 2, we tested the indirect effect of perceived weight discrimination on the relation between obesity and change in depressive symptoms while controlling for health and health behavior variables. Once again, these analyses confirmed that perceived weight discrimination significantly mediated the relation between obesity and change in depressive symptoms, explaining approximately 30% of this association (see Table S2 in the online supplemental materials). Similarly, our supplementary analyses confirmed that weight discrimination mediated the association between continuous BMI and depressive symptoms (explaining 54.2% of this link) and mediated the link between Class II and Class III obesity and clinically significant depression (explaining 38.3% of the association), as shown in Tables S8 and S9 of the online supplemental materials.

#### Additional mediation analysis

In our main analyses for Study 3, we combined perceived weight discrimination scores measured at baseline and follow-up. However, further analyses also showed that obesity at baseline predicted increases in weight discrimination from baseline to follow-up, and this increase explained changes in depressive symptoms over time. More specifically, in unadjusted analyses obesity Classes I, II, and III showed a strong graded associated with increases in weight discrimination from baseline to follow-up (Class I: *OR* = 5.39, 95% CI = 3.60–8.07; Class II: *OR* = 8.07, 95% CI = 4.92–13.23; Class III: *OR* = 24.47. 95% CI = 13.06–45.84). In analyses adjusting for baseline weight discrimination and covariates, we found that only obesity Class III predicted longitudinal increases in depressive symptoms (total effect: β = .220, *p* < .05). Including changes in weight discrimination between baseline and follow-up in this model explained 25.5% of the longitudinal association between obesity Class III and subsequent changes in depressive symptoms (indirect effect: β = .056, *p* < .05). Thus, the association between obesity and longitudinal change in depressive symptoms is in part explained by experiencing weight discrimination when changes in perceived weight discrimination over time are examined as a mediator.

### Additional Analyses

#### Gender

Because women may be judged more critically than men because of their weight, we examined gender differences in each of the key study variables (i.e., obesity, weight discrimination, depressive symptoms) and tested whether gender moderated the relation between perceived weight discrimination and depressive symptoms. We did this by including Gender × Perceived Weight Discrimination interactions in the earlier reported regression models for Studies 1 through 3 and examined whether this explained further variance in depressive symptoms.

Across the three studies, we found little evidence that rates of obesity differed between men and women. However, women showed larger increases in depressive symptoms than men in all studies, as shown in Table S10 of the online supplemental materials. Women were also more likely than were men to experience weight-based discrimination in Studies 2 and 3. In Study 3 (MIDUS), women experienced a particularly increased risk of weight discrimination (*OR* = 2.207, 95% CI = 1.750–2.784, *p* < .01) and depressive symptoms (β = .167, *SE* = .031, *p* < .01), potentially pointing to a gender difference in the mediating role of weight discrimination in that study.

There was no evidence that gender moderated the prospective association between perceived weight discrimination and depressive symptoms in Studies 1 and 2 (*p*s > .05). In Study 3, we identified a significant interaction that indicated perceived weight discrimination was more closely linked to change in depression among women. Supplementary mediation analyses showed that while obesity was linked to higher rates of perceived weight discrimination in both men and women, discrimination only acted as a pathway from obesity (Classes II and II) to depressive symptoms for women in Study 3 (explaining 43% of this association, see Table S11 of the online supplemental materials).

## General Discussion

We used three large samples of predominantly White U.S. and U.K. adults to test the hypothesis that experiencing weight-based discrimination mediates the prospective effect of obesity on depressive symptoms. In line with previous research (Preiss et al., 2013; Vogelzangs et al., 2010), we found consistent evidence that obesity (Classes II and III) was associated with increases in depressive symptoms over several years. Moreover, across all three samples the prospective association between obesity and depressive symptoms was in part explained by perceived weight discrimination; adults with obesity were more likely to report experiencing weight-based discrimination, which in turn predicted increases in depressive symptoms over time. On average, perceived weight discrimination was linked to an increase in depressive symptoms (0.16SD change), and on average explained 31% of the total effect of obesity Classes II and III on depressive symptoms.

The results of the present research are consistent with previous cross-sectional findings linking the experience of weight-based discrimination with impaired well-being and depressive symptoms (Chen et al., 2007; Jackson et al., 2015a). However, the present work is the first to show that there is a prospective association between perceived weight-based discrimination and increased depressive symptoms. To date, there has also been little research explaining potential mechanisms linking heavier body weight to longitudinal increases in depressive symptoms (Preiss et al., 2013; Remigio-Baker et al., 2014); our findings suggest that among U.S. and U.K. adults, perceived weight-based discrimination may be an important factor explaining this link. In Study 3, we observed that the effects on depressive symptoms of experiencing weight-based discrimination were more detrimental to women than to men, but this finding was not observed in either Study 1 or Study 2, so the replicability of this gender effect is unclear and warrants further attention.

Because of the observational nature of the present work, we cannot make strong claims about the causal influence that perceived weight discrimination has on the development of depressive symptoms. However, experimental work suggests that experiencing weight-based stigma increases negative affect (Himmelstein, Incollingo Belsky, & Tomiyama, 2015; Schvey, Puhl, & Brownell, 2011), and the present work adds to this emerging literature. Moreover, a number of theoretical models suggest that experiencing weight discrimination is likely to be stressful and may reduce self-worth (Crocker et al., 1993; Sikorski et al., 2015; Tomiyama, 2014), both of which are likely to increase depressive symptoms. Obesity is viewed negatively by large proportions of society and realizing that one is part of a stigmatized social group is likely to be psychologically distressing (Hunger & Major, 2015; Hunger, Major, Blodorn, & Miller, 2015). Experiencing weight discrimination may therefore reinforce negative beliefs about how a person with obesity believes they are viewed by others. Understanding the pathways by which experiencing weight-based discrimination is associated with increased depressive symptoms will now be important. Experiencing weight-based discrimination could also contribute to depressive symptoms by limiting employment opportunities, increasing body dissatisfaction (Wardle, Waller, & Rapoport, 2001), internalization of weight stigma (Durso & Latner, 2008), damaging self-esteem (Myers & Rosen, 1999) and/or by increasing feelings of loneliness (Lewis et al., 2011). Regardless of the pathways by which experiencing weight-based discrimination is associated with depressive symptoms, challenging discrimination based on weight will now be important and policies which challenge the derogation of persons with obesity or outline the damaging effects of weight stigma may be ways of achieving this.

### Limitations and Future Directions

Our focus in the present work was on middle age and older adulthood, so we do not know whether the same pattern of results would be observed among younger adults. Given that experiencing weight-based and other forms of discrimination have been associated with adverse health outcomes among younger age groups (Puhl & Heuer, 2009; Schmitt, Branscombe, Postmes, & Garcia, 2014; Wott & Carels, 2010) and obesity may be stigmatised most among younger age groups (Hebl et al., 2008), weight based discrimination may also play a role in explaining the link between obesity and depression in younger age groups. However, further work is now needed to test whether this process holds among younger adults. Further work would also benefit from considering the importance of personality variables when considering perceived weight discrimination and depressive symptoms, as it is plausible that factors such as neuroticism may increase the likelihood that a person perceives an experience as discriminatory and/or exacerbate the damaging psychological effects of discrimination. It should be noted that associations between experiencing discrimination and mental health in other studies tend to be robust, irrespective of adjusting for personality characteristics (Lewis, Cogburn, & Williams, 2015). A limitation of the present work was that we did not have very large numbers of participants with Class II and III obesity in each study, although we still observed consistent findings across studies and when BMI was used as a continuous predictor rather than weight status categories. Our samples also predominantly consisted of White participants and the lack of racial diversity could have influenced our results. It is therefore not clear whether experiencing weight discrimination is prospectively linked to increased depressive symptoms among other ethnic groups. Some final limitations concern Study 3: Because of practical constraints, only self-reported BMI data were available, and the measure of perceived weight discrimination was derived from participants’ reports of being discriminated against because of their size more generally (e.g., weight or height), as opposed to only their weight.

### Conclusions

In U.S. and U.K. samples, the prospective association between obesity and increases in depressive symptoms in adulthood may in part be explained by perceived weight discrimination.

## Supplementary Material

10.1037/hea0000426.supp

## Figures and Tables

**Table 1 tbl1:** Basic Demographic Characteristics and Descriptive Statistics for Participants in Studies 1, 2, and 3

	Study 1: ELSA (*N* = 6,000)			Study 2: HRS (*N* = 9,908)			Study 3: MIDUS (*N* = 4,378)		
Variable	*M*	*%*	*SD*	*M*	*%*	*SD*	*M*	*%*	*SD*
Age (years)	64.75		8.60	66.97		9.72	46.68		12.45
Female (%)		55.4			60.1			53.2	
White (%)		97.8			85.2			93.8	
BMI baseline (kg/m^2^)	28.29		5.17	29.39		5.83	26.62		5.16
Weight status (%)									
BMI ≤ 25 kg/m^2^		26.60			22.88			41.69	
Overweight		42.13			36.97			37.62	
Class I obese		21.30			24.60			13.98	
Class II obese		7.00			10.40			4.66	
Class III obese		2.97			5.15			2.06	
Depressive symptoms (baseline)	1.21^a^		1.78	1.69^b^		2.09	.70^c^		1.83
Depressive symptoms (follow-up)	1.21^a^		1.78	1.78^b^		2.13	.61^c^		1.72
*Note.* ELSA = English Longitudinal Study of Ageing; HRS = Health and Retirement Study; MIDUS = Midlife in the United States.									
^a^ Score ranging from 0 to 8, with higher scores indicating greater depressive symptoms. ^b^ Score ranging from 0 to 9, with higher scores indicating greater depressive symptoms. ^c^ Score ranging from 0 to 7, with higher scores indicating greater depressive symptoms.									

**Table 2 tbl2:** Percentage of Participants Reporting Experiencing Weight-Based Discrimination by Weight Status in Studies 1, 2, and 3

	Study 1: ELSA (*N* = 6,000)^a^		Study 2: HRS (*N* = 9,908)^b^		Study 3: MIDUS (*N* = 4,378)^c^	
Weight status	%	(*n/*total)	%	(*n/*total)	%	(*n/*total)
Normal weight (BMI < 25 kg/m^2^)	.9	(14/1,596)	1.9	(42/2,268)	4.9	(89/1,825)
Overweight	.9	(22/2,528)	2.5	(91/3,663)	8.4	(138/1,647)
Class I obese	5.9	(75/1,278)	9.1	(221/2,437)	21.2	(130/612)
Class II obese	20.5	(86/420)	20.8	(214/1,030)	38.7	(79/204)
Class III obese	32.6	(58/178)	36.5	(186/510)	58.9	(53/90)
*Note.* ELSA = English Longitudinal Study of Ageing; HRS = Health and Retirement Study; MIDUS = Midlife in the United States.						
^a^ Perceived weight discrimination among those reporting experiences of discrimination attributable to weight in the 2008/2009 wave of ELSA. ^b^ Perceived weight discrimination among those reporting experiences of discrimination attributable to weight in the 2006/2008 wave of HRS. ^c^ Perceived weight discrimination among those reporting experiences of discrimination attributable to weight/height in 1995/1996 or 2004/2005 waves of MIDUS.						

**Table 3 tbl3:** Mediation Models of the Indirect Effect of Obesity on Changes in Depressive Symptoms Through Perceived Weight Discrimination in Study 1 (ELSA; N = 6,000)

Analyses	Point estimate	*SE*	95% CI	Effect ratio
Class III obesity				
Weight Status → Discrimination (IV to mediator, path *a*)	3.892**	.320		
Discrimination → Depression (mediator to DV, path *b*)	.188**	.059		
Weight Status → Depression (total effect, path *c*)	.278**	.068		
Weight Status → Depression (direct effect, path *c′*)	.220**	.070		
Weight Status → Depression (indirect effect, path *a* × *b*)	.057**	.019	[.020−.095]	.206
Class II obesity				
Weight Status → Discrimination (IV to mediator, path *a*)	3.321**	.298		
Discrimination → Depression (mediator to DV, path *b*)	.188**	.059		
Weight Status → Depression (total effect, path *c*)	.197**	.047		
Weight Status → Depression (direct effect, path *c′*)	.161**	.048		
Weight Status → Depression (indirect effect, path *a* × *b*)	.036**	.012	[.013−.059]	.181
Class I obesity				
Weight Status → Discrimination (IV to mediator, path *a*)	2.021**	.297		
Discrimination → Depression (mediator to DV, path *b*)	.188**	.059		
Weight Status → Depression (total effect, path *c*)	.031	.032		
Weight Status → Depression (direct effect, path *c*′)	.021	.032		
Weight Status → depression (indirect effect, path *a* × *b*)				
*Note*. Models use *z* scores for depressive symptoms as the outcome variable. Models are adjusted for baseline depressive symptoms, age, age^2^, gender, ethnicity (White vs. other), educational attainment, marital status (married, cohabiting, other) and employment categories (employed/self-employed, unemployed, homemaker, retired, permanently sick or disabled). ELSA = English Longitudinal Study of Ageing; IV = independent variable; DV = dependent variable.				
** *p* < .01.				

**Table 4 tbl4:** Mediation Models of the Indirect Effect of Obesity on Changes in Depressive Symptoms Through Perceived Weight Discrimination in Study 2 (HRS; N = 9,908)

Analyses	Point estimate	*SE*	95% CI	Effect ratio
Class III obesity				
Weight Status → Discrimination (IV to mediator, path *a*)	3.289**	.186		
Discrimination → Depression (mediator to DV, path *b*)	.141**	.033		
Weight Status → Depression (total effect, path *c*)	.107**	.040		
Weight Status → Depression (direct effect, path *c′*)	.061	.042		
Weight Status → Depression (indirect effect, path *a* × *b*)	.046**	.011	[.024−.069]	.433
Class II obesity				
Weight Status → Discrimination (IV to mediator, path *a*)	2.612**	.177		
Discrimination → Depression (mediator to DV, path *b*)	.141**	.033		
Weight Status → Depression (total effect, path *c*)	.067*	.031		
Weight Status → Depression (direct effect, path *c′*)	.041	.032		
Weight Status → Depression (indirect effect, path *a* × *b*)	.026**	.006	[.013−.038]	.389
Class I obesity				
Weight Status → Discrimination (IV to mediator, path *a*)	1.732**	.173		
Discrimination → Depression (mediator to DV, path *b*)	.141**	.033		
Weight Status → Depression (total effect, path *c*)	.053*	.024		
Weight Status → Depression (direct effect, path *c′*)	.043	.024		
Weight Status → Depression (indirect effect, path *a* × *b*)	.011**	.003	[.005−.016]	.197
*Note*. Models use *z* scores for depressive symptoms outcome variable. Models are adjusted for baseline depressive symptoms, age, age^2^, gender, ethnicity (White vs. other), educational attainment, marital status (married, separated/divorced, widowed, never married) and employment categories (employed, unemployed, homemaker, retired, temporary leave, disabled). HRS = Health and Retirement Study; IV = independent variable; DV = dependent variable.				
* *p* < .05. ** *p* < .01.				

**Table 5 tbl5:** Mediation Models of the Indirect Effect of Obesity on Changes in Depressive Symptoms Through Perceived Weight Discrimination in Study 3 (MIDUS; N = 4,378)

Analyses	Point estimate	*SE*	95% CI	Effect ratio
Class III obesity				
Weight Status → Discrimination (IV to mediator, path *a*)	3.455**	.259		
Discrimination → Depression (mediator to DV, path *b*)	.152**	.048		
Weight Status → Depression (total effect, path *c*)	.293*	.101		
Weight status → Depression (direct effect, path *c*′)	.212	.104		
Weight Status → Depression (indirect effect, path *a* × *b*)	.081**	.026	[.028−.132]	.273
Class II obesity				
Weight Status → Discrimination (IV to mediator, path *a*)	2.751**	.193		
Discrimination → Depression (mediator to DV, path *b*)	.152**	.048		
Weight Status → Depression (total effect, path *c*)	.147*	.069		
Weight Status → Depression (direct effect, path *c*′)	.094	.071		
Weight Status → Depression (indirect effect, path *a* × *b*)	.052**	.017	[.018−.086]	.356
Class I obesity				
Weight Status → Discrimination (IV to mediator, path *a*)	2.040**	.157		
Discrimination → Depression (mediator to DV, path *b*)	.152**	.048		
Weight Status → Depression (total effect, path *c*)	.001	.044		
Weight Status → Depression (direct effect, path *c*′)	−.027	.045		
Weight Status → Depression (indirect effect, path *a* × *b*)				
*Note*. Models use *z* scores for depressive symptoms outcome variable. Models are adjusted for age, age^2^, gender, ethnicity (White vs. other), educational attainment, marital status (married, separated, divorced, widowed, never married), and employment categories (employed, self-employed, unemployed, laid off, homemaker, student, retired, on leave, permanently disabled, other). MIDUS = Midlife in the United States; IV = independent variable; DV = dependent variable.				
* *p* < .05. ** *p* < .01.				
