# Supplementary material for: Perceived Weight Discrimination Mediates the Prospective Relation Between Obesity and Depressive Symptoms in U.S. and U.K. Adults
Source: Health Psychol. 2016 Oct 17;36(2):112–21. doi: 10.1037/hea0000426 (PMC5267562; doi:10.1037/hea0000426)
Supplement: Supplementary file 1 [file zg1-3341_HEA-2016-3809_SUPPL.zip › zg1011163341so1.doc]

**Supplemental Materials**

**Perceived Weight Discrimination Mediates the Prospective Relation Between Obesity and Depressive Symptoms in US and UK Adults**

**by E. Robinson et al., 2016, *Health Psychology***

**http://dx.doi.org/10.1037/hea0000426**

**Table S1.** *Percentage of Participants Reporting Experiencing Clinically Significant Depression at Follow-up by Weight Status in Studies 1-3.*

|  | Study 1/ ELSA^a^  *N* = 6,000 | Study 2 / HRS^b^  *N* = 9,908 | Study 3 /MIDUS^c^  *N* = 4,378 |
| --- | --- | --- | --- |
|  | % (*N* of total) | % (*N* of total) | % (*N* of total) |
| Total sample | 11.0 (659/6000) | 17.5 (1733/9908) | 10.2 (445/4378) |
| Normal weight  (BMI < 25 kg/m^2^) | 10.3 (164/1596) | 14.8 (336/2268) | 10.4 (189/1825) |
| Overweight | 9.7 (245/2528) | 16.5 (604/3663) | 8.7 (144/1647) |
| Class I obese | 10.3 (131/1278) | 17.6 (429/2437) | 9.5 (58/612) |
| Class II obese | 18.8 (79/420) | 21.6 (222/1030) | 16.7 (34/204) |
| Class III obese | 22.5 (40/178) | 27.8 (142/510) | 22.2 (20/90) |

^a^ Clinically significant depression: those scoring ≥ 4 on the 8-item Center for Epidemiology Depression Scale (CES-D) administered in the 2012/2013 wave of ELSA.

^b^ Clinically significant depression: those scoring ≥ 4 on the 9-item Center for Epidemiology Depression Scale (CES-D) administered in the 2010/2012 wave of HRS.

^c^ Clinically significant depression: those meeting the World Health Organization Composite International Diagnostic Interview-Short Form (CITI-SD) criteria for depression in the 2004/2005 wave of MIDUS.

**Table S2.** *Mediation Models of the Indirect Effect of Obesity on Longitudinal Changes in Depressive Symptoms through Weight Discrimination in Studies 1-3 in Models including Health Status & Health Behavior as Covariates.*

|  | Study 1/ ELSA  *N* = 6,000 | | | Study 2 / HRS  *N* = 9,908 | | | Study 3 /MIDUS  *N* = 4,378 | |
| --- | --- | --- | --- | --- | --- | --- | --- | --- |
|  | b | SE | | b | SE | | b | SE |
| **Class III Obesity** | | | | | | | | |
| Weight status ->  discrimination  *(IV to mediator, path a)* | 3.611** | .331 | 3.301** | | | .187 | 3.38** | .262 |
| Discrimination -> depression  *(mediator to DV, path b)* | .162** | .058 | .141** | | | .033 | .147** | .048 |
| Weight status -> depression  *(total effect, path c)* | .159* | .068 | .106** | | | .040 | .293** | .101 |
| Weight status -> depression  *(direct effect, path c’)* | .111 | .071 | .059 | | | .042 | .216* | .104 |
| Weight status -> depression  (*indirect effect, path a× b*) | .048** | .018 | .047** | | | .011 | .077** | .026 |
| **Class II Obesity** | | | | | | | | |
| Weight status ->  discrimination  *(IV to mediator, path a)* | 3.15** | .303 | 2.621** | | | .178 | 2.692** | .195 |
| Discrimination -> depression  *(mediator to DV, path b)* | .162** | .058 | .141** | | | .033 | .147** | .048 |
| Weight status -> depression  *(total effect, path c)* | .113* | .047 | .064* | | | .031 | .152* | .069 |
| Weight status -> depression  *(direct effect, path c’)* | .084 | .048 | .038 | | | .032 | .102 | .071 |
| Weight status -> depression  (*indirect effect, path a× b*) | .030** | .011 | .026** | | | .006 | .050** | .017 |
| **Class I Obesity** | | | | | | | | |
| Weight status -> discrimination  *(IV to mediator, path a)* | 1.91** | .299 | 1.74** | | | .174 | 2.002** | .158 |
| Discrimination -> depression  *(mediator to DV, path b)* | .162** | .058 | .141** | | | .033 | .147** | .048 |
| Weight status -> depression  *(total effect, path c)* | -.005 | .032 | .050* | | | .024 | .007 | .044 |
| Weight status -> depression  *(direct effect, path c’)* | -.013 | .032 | .039 | | | .024 | -.020 | .045 |
| Weight status -> depression  (*indirect effect, path a× b*) | – | – | .011** | | | .002 | – | – |

*Note*. Models use z-scores for depressive symptoms outcome variable.

Models are adjusted for baseline depressive symptoms, age, age-squared, sex, ethnicity (white vs. other), educational attainment, marital status, employment categories, current smoking, alcohol consumption, physical activity levels, and presence of chronic illness.

**p*<.05, ***p*<.01.

**Table S3.** *Mediation Models of the Indirect Effect of BMI on Changes in Depressive Symptoms through Perceived Weight Discrimination in Study 1 (ELSA; N = 6,000).*

|  | Point Estimate | SE | 95% CI Lower ; Upper | | Effect ratio | |
| --- | --- | --- | --- | --- | --- | --- |
|  | | | | | | |
| BMI -> discrimination  *(IV to mediator, path a)* | .205** | .011 |  |  |  | |
| Discrimination -> depression  *(mediator to DV, path b)* | .202** | .058 |  |  |  |  |
| BMI -> depression  *(total effect, path c)* | .011** | .002 |  |  |  | |
| BMI -> depression  *(direct effect, path c’)* | .008** | .002 |  |  |  | |
| BMI -> depression  *(indirect effect, path a× b)* | .002** | .0001 | [.001 ; .004] | | .229 | |

*Note*. Models use z-scores for depressive symptoms as the outcome variable. Models are adjusted for baseline depressive symptoms, age, age-squared, gender, ethnicity (white vs. other), educational attainment, marital status (married, cohabiting, other) and employment categories (employed/self-employed, unemployed, homemaker, retired, permanently sick or disabled). **p*<.05, ***p*<.01.

**Table S4.** *Perceived Weight Discrimination and Changes in Levels of Clinically Significant Depression in Studies 1-3.*

|  | Study 1/ ELSA^a^  *N* = 6,000 | Study 2 / HRS^b^  *N* = 9,908 | Study 3 /MIDUS^c^  *N* = 4,378 |
| --- | --- | --- | --- |
|  | OR (95% CI) | OR (95% CI) | OR (95% CI) |
| Weight discrimination | 1.51* (1.04-2.19) | 1.50** (1.22-1.84) | 1.45* (1.01-2.09) |

*Note*. Models are adjusted for weight categories (BMI ≤ 25, overweight, obese I, obese II, obese III) and depression at baseline and age, age-squared, gender, ethnicity (white vs. other), educational attainment, marital status and employment categories.

**p*<.05, ***p*<.01.

**Table S5.** *Mediation Models of the Indirect Effect of Obesity on Changes in Clinically Significant Depression through Perceived Weight Discrimination in Study 1 (ELSA; N = 6,000).*

|  | Point Estimate | SE | 95% CI Lower ; Upper | Effect ratio |
| --- | --- | --- | --- | --- |
| **Class III Obesity** | | | | |
| Weight status -> depression  *(indirect effect, path a× b)* | .016* | .006 | [.004 ; .029] | .258 |
| **Class II Obesity** | | | | |
| Weight status -> depression  *(indirect effect, path a× b)* | .010** | .004 | [.002 ; .018] | .187 |
| **Class I Obesity** | | | | |
| Weight status -> depression  *(indirect effect, path a× b)* | – | – | – | – |

*Note*. Models are adjusted for depression at baseline, age, age-squared, gender, ethnicity (white vs. other), educational attainment, marital status (married, cohabiting, other) and employment categories (employed/self-employed, unemployed, homemaker, retired, permanently sick or disabled).

**p*<.05, ***p*<.01.

**Table S6.** *Mediation Models of the Indirect Effect of BMI on Changes in Depressive Symptoms through Perceived Weight Discrimination in Study 2 (HRS; N = 9,908).*

|  | Point Estimate | SE | 95% CI Lower ; Upper | | Effect ratio |
| --- | --- | --- | --- | --- | --- |
|  | | | | | |
| BMI -> discrimination  *(IV to mediator, path a)* | .157** | .006 |  |  |  |
| Discrimination -> depression  *(mediator to DV, path b)* | .135** | .033 |  |  |  |
| BMI -> depression  *(total effect, path c)* | .005** | .001 |  |  |  |
| BMI -> depression  *(direct effect, path c’)* | .003 | .002 |  |  |  |
| BMI -> depression  *(indirect effect, path a× b)* | .002** | .0004 | [.001 ; .003] | | .386 |

*Note*. Models use z-scores for depressive symptoms outcome variable. Models are adjusted for baseline depressive symptoms, age, age-squared, gender, ethnicity (white vs. other), educational attainment, marital status (married, separated/divorced, widowed, never married) and employment categories (employed, unemployed, homemaker, retired, temporary leave, disabled). **p*<.05, ***p*<.01.

**Table S7.** *Mediation Models of the Indirect Effect of Obesity on Changes in Clinically Significant Depression through Perceived Weight Discrimination in Study 2 (HRS; N = 9,908).*

|  | Point Estimate | SE | 95% CI Lower ; Upper | Effect ratio |
| --- | --- | --- | --- | --- |
| **Class III Obesity** | | | | |
| Weight status -> depression  *(indirect effect, path a× b)* | .019** | .005 | [.010 ; .029] | .273 |
| **Class II Obesity** | | | | |
| Weight status -> depression  *(indirect effect, path a× b)* | .011** | .003 | [.006 ; .016] | .304 |
| **Class I Obesity** | | | | |
| Weight status -> depression  *(indirect effect, path a× b)* | .004** | .001 | [.002 ; .007] | .216 |

*Note*. Models are adjusted for depression at baseline, age, age-squared, gender, ethnicity (white vs. other), educational attainment, marital status (married, separated/divorced, widowed, never married) and employment categories (employed, unemployed, homemaker, retired, temporary leave, disabled).

**p*<.05, ***p*<.01.

**Table S8.** *Mediation Models of the Indirect Effect of BMI on Changes in Depressive Symptoms through Perceived Weight Discrimination in Study 3 (MIDUS; N = 4,378).*

|  | Point Estimate | SE | 95% CI Lower ; Upper | | Effect ratio | |
| --- | --- | --- | --- | --- | --- | --- |
|  | | | | | | |
| BMI -> discrimination  *(IV to mediator, path a)* | .177** | .010 |  |  |  | |
| Discrimination -> depression  *(mediator to DV, path b)* | .164** | .048 |  |  |  |  |
| BMI -> depression  *(total effect, path c)* | .006* | .003 |  |  |  | |
| BMI -> depression  *(direct effect, path c’)* | .003 | .003 |  |  |  | |
| BMI -> depression  *(indirect effect, path a× b)* | .003** | .001 | [.001 ; .005] | | .542 | |

*Note*. Models use z-scores for depressive symptoms outcome variable. Models are adjusted for age, age-squared, gender, ethnicity (white vs. other), educational attainment, marital status (married, separated, divorced, widowed, never married) and employment categories (employed, self-employed, unemployed, laid off, homemaker, student, retired, on leave, permanently disabled, other).

**p*<.05, ***p*<.01.

**Table S9.** *Mediation Models of the Indirect Effect of Obesity on Changes in Clinically Significant Depression through Perceived Weight Discrimination in Study 3 (MIDUS; N = 4,378).*

|  | Point Estimate | SE | 95% CI Lower ; Upper | Effect ratio |
| --- | --- | --- | --- | --- |
| **Class III Obesity** | | | | |
| Weight status -> depression  *(indirect effect, path a× b)* | .029** | .008 | [.013 ; .045] | .351 |
| **Class II Obesity** | | | | |
| Weight status -> depression  *(indirect effect, path a× b)* | .019** | .005 | [.008 ; .030] | .415 |
| C**lass I Obesity** | | | | |
| Weight status -> depression  *(indirect effect, path a× b)* | – | – | – | – |

*Note*. Models are adjusted for age, age-squared, gender, ethnicity (white vs. other), educational attainment, marital status (married, separated, divorced, widowed, never married) and employment categories (employed, self-employed, unemployed, laid off, homemaker, student, retired, on leave, permanently disabled, other).

**p*<.05, ***p*<.01.

**Table S10.** *Association Between Female Gender and Obesity, Perceived Weight Discrimination and Depressive Symptoms in Studies 1-3.*

|  | Study 1/ ELSA  *N* = 6,000 | Study 2 / HRS  *N* = 9,908 | Study 3 /MIDUS  *N* = 4,378 |
| --- | --- | --- | --- |
|  | OR / B  (95% CI / SE) | OR / B  (95% CI / SE) | OR / B  (95% CI / SE) |
| Obesity classes I/II/III^a^ | 1.131*  (1.004-1.271) | .995  (.909-1.089) | .923  (.787-1.084) |
| Weight discrimination^b^ | 1.040  (.767-1.410) | 1.51**  (1.250-1.825) | 2.207**  (1.750-2.784) |
| Depressive symptoms (follow-up)^c^ | .085**  (.024) | .059**  (.018) | .167**  (.031) |

*Note*. Each row represents a separate analysis of gender differences.

^a^Odds ratios are presented. Models are adjusted for depressive symptoms at baseline and age, age-squared, gender, ethnicity (white vs. other), educational attainment, marital status and employment categories.

^b^Odds ratios are presented. Models are adjusted for baseline depressive symptoms and weight categories (BMI ≤ 25, overweight, obese I, obese II, obese III) and age, age-squared, gender, ethnicity (white vs. other), educational attainment, marital status and employment categories.

^c^OLS coefficients are presented. Models use z-scores for depressive symptoms outcome variable. Models are adjusted for baseline depressive symptoms and weight categories (BMI ≤ 25, overweight, obese I, obese II, obese III) and age, age-squared, gender, ethnicity (white vs. other), educational attainment, marital status and employment categories.

**p*<.05, ***p*<.01.

**Table S11.** M*ediation Models of the Indirect Effect of Obesity on Longitudinal Changes in Depressive Symptoms through Weight Discrimination for Males and Females in Study 3 (MIDUS).*

|  | |  | | Male  *N* = 2,051 | | | | Female  *N* = 2,327 | | | |
| --- | --- | --- | --- | --- | --- | --- | --- | --- | --- | --- | --- |
|  | |  | | b | SE | | | b | | SE | |
| **Class III Obesity** |  | |  | | |  |  | |  | |  |
| Weight status -> discrimination  *(IV to mediator, path a)* |  | | 3.228** | | | .444 | 3.568** | | .316 | |  |
| Discrimination -> depression  *(mediator to DV, path b)* |  | | -.036 | | | .066 | .240** | | .069 | |  |
| Weight status -> depression  *(total effect, path c)* |  | | -.120 | | | .153 | .437** | | .137 | |  |
| Weight status -> depression |  | | -.103 | | | .156 | .305** | | .142 | |  |
| *(direct effect, path c’)* |  | |  | | |  |  | |  | |  |
| Weight status -> depression  (*indirect effect, path a× b*) |  | | – | | | – | .132** | | .041 | |  |
| **Class II Obesity** |  | |  | | |  |  | |  | |  |
| Weight status -> discrimination  *(IV to mediator, path a)* |  | | 1.807** | | | .345 | 3.324** | | .246 | |  |
| Discrimination -> depression  *(mediator to DV, path b)* |  | | -.036 | | | .066 | .240** | | .069 | |  |
| Weight status -> depression  *(total effect, path c)* |  | | .044 | | | .093 | .198* | | .099 | |  |
| Weight status -> depression |  | | .050 | | | .093 | .088 | | .104 | |  |
| *(direct effect, path c’)* |  | |  | | |  |  | |  | |  |
| Weight status -> depression  (*indirect effect, path a× b*) |  | | – | | | – | .110** | | .034 | |  |
| **Class I Obesity** |  | |  | | |  |  | |  | |  |
| Weight status -> discrimination  *(IV to mediator, path a)* |  | | 1.394** | | | .259 | 2.344** | | .198 | |  |
| Discrimination -> depression  *(mediator to DV, path b)* |  | | -.036 | | | .066 | .240** | | .069 | |  |
| Weight status -> depression  *(total effect, path c)* |  | | -.066 | | | .054 | .022 | | .069 | |  |
| Weight status -> depression |  | | -.062 | | | .054 | -.039 | | .071 | |  |
| *(direct effect, path c’)* |  | |  | | |  |  | |  | |  |
| Weight status -> depression  (*indirect effect, path a× b*) |  | | – | | | – | – | | – | |  |

*Note*. Models use z-scores for depressive symptoms outcome variable.

Models are adjusted for age, age-squared, sex, ethnicity (white vs. other), marital status (married, separated, divorced, widowed, never married) and employment categories (employed, self-employed, unemployed, laid off, homemaker, student, retired, on leave, permanently disabled, other).

**p*<.05, ***p*<.01.
